# Dr. Gardette and His Proposition, Once More

**Published:** 1851-07

**Authors:** 


### EDITORIAL DEPARTMENT.

*Dr. Gardette and his Proposition, once more.*—In the last number of our Journal, we published a letter from Dr. Gardette, of Philadelphia, to the editor of the American Journal of Medical Sciences, suggesting the addition of a professorship of dental surgery to the several Medical Faculties, as a substitute for any scheme of separate collegiate education for dentists. In our comments, we endeavored to be kind and respectful to Dr. Gardette. He had an indisputable right to make the suggestions he did, and there was nothing in his language to which, personally, we could take offence. But the suggestion itself we reprobated, as we believed it deserved. We considered it altogether absurd, as any attempt to act upon it would be impracticable. Dr. G. seems to be much affronted at our remarks, and he has rejoined in the Medical Examiner in a tone, which, as his friend, we regret. Though he had been unwise, there was no necessity to be offended. He says he has done his part in this matter "with no more vanity, interest and ambition about its adoption, than became a plain man," &c.! How much of vanity, interest and ambition Dr. G. may think becoming, it is not for us to determine.

After a careful examination of Dr. Gardette's rejoinder, we cannot perceive that we have done him injustice. We reprinted the whole of his letter, and our readers shall be our judges, whether we ascribed to the writer a single expression, or opinion, not plainly justified by his own words. We attempted to show that the working of the scheme would be impracticable. In this we are pleased to find that Dr. G. now agrees with us. He now declares, that he never intended to sketch the manner in which the scheme was to be carried out. That, he left entirely to the wisdom of the Trustees and Professors of Medical Colleges, who, from their familiarity with the subject—and their long manifested interest in dentistry, would, doubtless, be able to do what Dr. G. himself dared not to undertake, and what we, in endeavoring to do, have produced only an absurdity, which Dr. G. cannot even look at without indignation.

In one respect, however, the Dr. is much plainer than before. He now tells us, that he does not expect practical nor mechanical dentistry to be taught in *his* colleges. These, he says, are to be taught by the private preceptor, "and it is there that careful, judicious operators will be made, whether they listen to lectures in medical or dental colleges."

The inuendo in the last paragraph is too plain to be misunderstood, and too unjust to be permitted to pass without notice. Dr. Gardette knows very well, that dental colleges do not expect to make dentists, by lectures merely. He knows that in the Baltimore College, which he mentions particularly, a great part of the means of the institution, and time of the instructors and students, are devoted to operations upon the mouth of the living subject, and the mechanical work incident thereto. He has himself examined operations performed by the pupils of the school, who learned in the institution to perform them, and he has been very decided in his expressions of satisfaction at what he saw. As to the fact, that careful and judicious operators may be made in private offices, we do not doubt, nor do we doubt, that careless and injudicious ones are made there. While the majority of those who live by dental operations are both careless and injudicious, it is likely that a majority of the students will be so too, and we should like to know, how the Trustees and Professors of medical colleges will distinguish between them. How many practitioners of dental surgery in Philadelphia, is Dr. Gardette in the habit of considering careful and judicious, and capable of instructing students? We leave him to answer the question. Dr. G. denies that he suggested, or intended, that dental students were to be thoroughly instructed in medicine. He considers such an interpretation of his language as evidence of consternation, producing confusion of ideas.

Where the confusion is, will be easily ascertained from the following quotation from Dr. Gardette's proposition. "*They* (the students) *should still be required to earn and receive a diploma with the title of M. D., as a guarantee of fitness to practice and a claim to confidence as dentist.*"

In his rejoinder, he writes, "*I have suggested no scheme according to which dental students are to be thoroughly instructed in all the branches of medicine. The abstracted professor, I must suppose, imagines these statements.*"

Now, how Dr. Gardette expects students to *earn* and *receive* the degree of doctor of medicine, without being instructed in all the branches of medicine, we confess we do not comprehend. Probably, in this respect too, Dr. G. does not pretend to prescribe the manner, but leaves the details to the wisdom of the Trustees and Faculties of medical colleges.

One thing is evident, that in Dr. G's scheme, the dental student is to have no excess of theoretical, to counterbalance his deficiency in practical, knowledge.

In this connection, Dr. Gardette become facetious. He thinks that we must have been much confused, indeed, when we termed his scheme "suicidal," and thinks we "mistook him for a dental college." Now we assure the Dr. that whatever mistakes we may have made about him, we never have erred so far as that. But we admit, that the degradation of dentistry by his hand would not be "suicidal" to the man who secretly despised it, and who felt the name of dentist a reproach.

Dr. Gardette sneers at us for assuming too much consequence for dental colleges. It does not become us to contest this question. Fortunately an arbiter is at hand, to whom Dr. G. cannot object. We will let him decide. Of Dr. Hullihen, Dr. Gardette says, "A gentleman who fulfils the duties of both medical practitioner and dentist with such distinguished abilities as to be an honor to both professions, has traced truthfully the origin of the neglect of which the dentist complains." Fortunately the same gentleman has traced truthfully the consequences of the institution of dental colleges, in the following language: *"It was not until the founding of this* (the Baltimore) *college—until medical science and mechanical skill were here first taught, both separately and combined, as the only plan to make an accomplished dental surgeon, that dental surgery became a science in spirit and in truth."*

Inasmuch as students of dental surgery, generally, do not avail themselves of the instruction given in dental colleges, Dr. Gardette infers, that if dental lectureships were established in medical colleges, they would be attracted to these institutions. But, if he were as well informed upon this subject as we are, he would think differently. If such lectureships existed in all the medical colleges in the United States, we are confident the lectures would not be listened to by twenty-five students of dentistry. To foresee the consequences, then, that would be likely to result from the establishment of such lectureships, requires not the gift of prophecy. A majority of the graduates who failed to obtain medical practice, and having no other means of subsistence than the avails of their profession, would naturally turn their attention to this specialty, and thus, in a short time, the whole country would be overrun with dental empirics of the lowest grade.

As an argument in favor of his scheme, Dr. Gardette says, "It is common with dentists of high standing, to require of their pupils that they attend medical lectures and take the degree of M. D.; and this is the case *even since the existence of dental colleges*." Without supposing for a moment, that Dr. G. made the above statement with a view of making a wrong impression upon the minds of his readers, the correctness of it must be regarded as exceedingly questionable. Touching the matter to which it refers, we presume, we are as well, if not better informed than Dr. G., and we have no hesitation in saying, that in nine cases out of ten, even among dentists of "high standing," no such requirement is made, and in the majority of cases where it is, it is not complied with. Within the last few years, several dentists of "high standing," have sent their pupils to dental colleges. In a number of other instances, students of dentistry, after having passed through Dr. Gardette's corriculum and graduated in medical colleges, have received the instructions of dental colleges, and frankly acknowledged afterwards, that they obtained more information there, in one session, than they had been able to do by the most diligent application in two years elsewhere.

There is one more paragraph of Dr. Gardette's paper which we are compelled to notice, though we do it with unfeigned reluctance. We cannot shut our eyes to what must be visible to all readers. It would be affectation to pretend not to understand the "animus" of the following sentence:

*"It has been stated to me that the Baltimore Dental College has been chiefly sustained thus far, in its feeble existence, by the talents and influence of its medical professors, and that one of these, who has been the main prop of the institution, has of late years designed to abandon it as an expensive, unsuccessful attempt."*

Who made this statement to Dr. Gardette we of course cannot tell. We trust he was somebody whose previous character for carefulness of speech will bear the deduction which must be made for the carelessness of this. As the faculty have never yet raised the question among themselves, as to the individual importance of its members, it might have been as well for others not to have troubled themselves about it. We certainly feel no disposition to undervalue the "talents and influence of the medical professors." We are glad that Dr. Gardette, even through contention, gives them proper credit. As he admits that we have done some service in the march of improvement, upon the whole, the faculty have escaped from his hands tolerably well. If it be true, that our medical colleagues have done more than we to sustain the school, we are glad of it. We have done what we could, and like the old Spartan who lost his election, we are consoled to know that the faculty possess at least two better men than ourselves.

In one thing, however, it is necessary to correct the Dr's informant. The students are not more attracted towards theoretical than practical study. The tendency is the other way. The infirmary and mechanical rooms are so attractive that it requires all the talent of our colleagues, and the stringent regulations of the school, to obtain sufficient consideration for the medical branches. The habits of dentists, such as dentists have been, and for the most part yet are, is to undervalue collateral scientific acquirements and make dentistry, as far as possible, a mechanical art. The present constitution of dental colleges tends to check this tendency. Let them be abolished, and let Dr. Gardette's scheme be tried instead, and suppose, for the sake of argument, that students of dentistry would avail themselves of the instruction of medical colleges, the consequence would be, that while a very few from offices of skillful men would attend the college lectures, a great many more from offices of ignorant pretenders, would do the same—none of them would be educated as physicians, very few as dentists, and all would be thrown upon the community with the accredited pretensions of graduates. The distinction between educated and uneducated, or trustworthy and unsafe dentists, would never be drawn—dentistry would not have a higher social position than now, and Dr. Gardette would find that in grasping at the shadow he had lost the substance.

With regard to the statement that one of the medical professors "who has been the main prop of the institution, has of late years designed to abandon it," it is necessary to say that Dr. Gardette has been entirely misinformed. We cannot pretend to say to which of the gentlemen Dr. G. alludes. We regard both of them as "props" to the institution, and we are happy to say, that unless some untoward accident should occur to remove them from this world of toil, they are likely to remain "props" for many years to come. So far from being discouraged with the undertaking, they both believe that the severity of the struggle is over, and the success of the school certain. That men like Dr. Gardette may disappoint their hopes and impede the progress of those who are toiling for the real good of the profession is possible, but the ultimate success of any undertaking of the kind in question, must depend upon the wants of community and its adaptedness to them. The want exists, and until something better than Dr. Gardette's proposition is offered, dental colleges are the only means adapted to supply the want.

In conclusion, we would direct the attention of our readers to an able and admirably written article, in another part of our Journal, by Dr. E. Townsend, of Philadelphia, showing the inexpediency and impracticability of Dr. Gardette's scheme.

